# Antibacterial Peptide-Based Gel for Prevention of Medical Implanted-Device Infection

**DOI:** 10.1371/journal.pone.0145143

**Published:** 2015-12-14

**Authors:** Mihaela Mateescu, Sébastien Baixe, Tony Garnier, Loic Jierry, Vincent Ball, Youssef Haikel, Marie Hélène Metz-Boutigue, Michel Nardin, Pierre Schaaf, Olivier Etienne, Philippe Lavalle

**Affiliations:** 1 Institut National de la Santé Et de la Recherche Médicale, Unité Mixte de Recherche-S 1121, Biomatériaux et Bioingénierie, Strasbourg, France; 2 Faculté de Chirurgie Dentaire, Université de Strasbourg, Strasbourg, France; 3 Institut Charles Sadron, Centre National de la Recherche Scientifique, Université de Strasbourg, Strasbourg, France; 4 Institut de Science des Matériaux de Mulhouse, Centre National de la Recherche Scientifique LRC 7228, Mulhouse, France; Academia Sinica, TAIWAN

## Abstract

Implanted medical devices are prone to infection. Designing new strategies to reduce infection and implant rejection are an important challenge for modern medicine. To this end, in the last few years many hydrogels have been designed as matrices for antimicrobial molecules destined to fight frequent infection found in moist environments like the oral cavity. In this study, two types of original hydrogels containing the antimicrobial peptide Cateslytin have been designed. The first hydrogel is based on alginate modified with catechol moieties (AC gel). The choice of these catechol functional groups which derive from mussel’s catechol originates from their strong adhesion properties on various surfaces. The second type of gel we tested is a mixture of alginate catechol and thiol-terminated Pluronic (AC/PlubisSH), a polymer derived from Pluronic, a well-known biocompatible polymer. This PlubisSH polymer has been chosen for its capacity to enhance the cohesion of the composition. These two gels offer new clinical uses, as they can be injected and jellify in a few minutes. Moreover, we show these gels strongly adhere to implant surfaces and gingiva. Once gelled, they demonstrate a high level of rheological properties and stability. In particular, the dissipative energy of the (AC/PlubisSH) gel detachment reaches a high value on gingiva (10 J.m^-2^) and on titanium alloys (4 J.m^-2^), conferring a strong mechanical barrier. Moreover, the Cateslytin peptide in hydrogels exhibited potent antimicrobial activities against *P*. *gingivalis*, where a strong inhibition of bacterial metabolic activity and viability was observed, indicating reduced virulence. Gel biocompatibility tests indicate no signs of toxicity. In conclusion, these new hydrogels could be ideal candidates in the prevention and/or management of periimplant diseases.

## Introduction

Biohydrogels have been developed in medicine for a broad scope of therapeutic applications. They are currently used for the release of biomacromolecules or drugs [1], for wound healing, or as a barrier for contact lenses or ocular surface injuries [2, 3]. Some of these gels have an intrinsic antimicrobial activity (like chitosan-based gels [4]), but more often drugs are added in the gel in order to enhance microbial activity [5]. Antibiotics are frequently introduced in the gels for prevention or treatment of infections. The properties of these gels allow release of therapeutic agents [6]. AntiMicrobial Peptides (AMPs) represent one type of antimicrobial agents that can be used to fight infections without the disadvantages of more conventional antibiotics, which may cause gastrointestinal or allergic reactions, or produce antibiotic-resistant bacterial strains. AMPs are natural molecules, which are part of the innate immune response and possess anti-inflammatory, antibacterial, antiviral and even anticancer activities [7, 8]. One such agent is human Cateslytin (CTL), the active sequence of Catestatin (CgA_352-372_) and corresponding to the sequence CgA_352-366_ (RSMRLSFRARGYGFR). Catestatin, is a naturally occurring potent inhibitor (IC50 200–400 nM) of catecholamine release, acting at the nicotinic cholinergic receptor [9]. This peptide also displays potent vasodepressor activity and appears to diminish early in the course of development of hypertension [10]. CTL is the active core sequence and displays highest nicotinic antagonism of catecholamine secretion [9]. CTL is also able to activate histamine release [11] and displays potent antimicrobial activities in the low micromolar range against bacteria, fungi, yeasts and Plasmodium [12, 13]. Time-lapse video microscopy showed arrest of fungal growth upon penetration of the rhodaminated peptide into a fungal filament [12]. Cateslytin was also identified in the stimulated secretions of human polymorphonuclear neutrophils [12]. All together, these properties show that CTL is a novel component of innate immunity.

In recent years, the efficiency of films functionalized with various AMPs has been tested. In particular, these coatings can be constructed using polyelectrolyte multilayer films [14–16]. Gels combining such molecules are promising solutions for coating a number of medical devices destined for human implantation [17]. A hyaluronic acid-based gel mixed with gentamycin has been assessed in rabbit and mouse models for its ability to limit postoperative infection following total joint arthroplasty [18]. Antibacterial wound gels can be applied to catheters used in peritoneal dialysis to prevent infection [19]. In oral implantology, bacteria are present in dental plaque and along with in the microgap between implant and prosthesis screws on implants. Specific pathogenic bacteria such as *Porphyromonas gingivalis* (Pg) can secrete virulent factors towards marginal gingiva, leading to periimplantitis. This chronic inflammation is characterized by a gingival inflammation and associated periimplant bone destruction [20]. Local or systemic antibiotic therapies are not able to decontaminate theses spaces [21] and may promote resistance and further impair treatment options. Sealing gels have been proposed to limit microleakage along the microgap of the connection, but their bioactivity is mainly based on chlorhexidine whose effects are limited in time, undergoing degradation in oral conditions [22–25].

Recently, catechol-based gels, inspired from the strong adhesive properties of mussel on mineral, metal and wood surfaces, have been developed for medical use [26], and represent compatible biohydrogels ideal for a moist environment like oral cavity. Catechol functional groups can be grafted on chitosan or alginate backbone, to form an alginate-chitosan or an alginate-catechol (AC) gel. Thiol-terminated Pluronic (PlubisSH) have been recently added to chitosan/catechol or hyaluronic acid/catechol polymers in order to enhance mechanical properties [27, 28]. However, these gels were built without any functionalization agent, like antibiotic or AMP.

Hence, we decided to develop a new injectable, adhesive and antimicrobial gel that could be employed on medical titanium surfaces, especially in area between dental implants and prosthesis. Alginate-Catechol/PlubisSH gels functionalized with the antimicrobial peptide CTL have been assessed in this study for their promising properties.

## Materials and Methods

### Materials

Alginate (medium viscosity), Pluronic F-127, NaIO_4,_ NaOH, PBS, were purchased from Sigma-Aldrich. CTL was obtained from Proteogenix (France). A 1 cm diameter biomedical Ti_6_Al_4_V bar was procured from ACNIS International Society (France). The bar was cut in several discs (1 cm diameter/1 cm thick), then polished using silicon carbide papers from ESCIL (France) with a decreasing grain-size (600, 800, 1200, 1600, 2000, 2400, 4000).

### Syntheses of AC and PlubisSH

Catechol-modified alginate (AC) and thiol-terminated Pluronic F-127 (PlubisSH) were prepared according to recently reported procedures (S1 Fig) [27, 28]. The degree of catechol substitution for AC was 15% and the degree of thiolation for PlubisSH was 65%.

### Hydrogels fabrication

We prepared two types of gels: alginate-catechol 1% (AC) and AC 0.5% / Pluronic bisSH18% (AC/PlubisSH). For the preparation of the first type of gel, we dissolved AC powder in a PBS solution at pH 7.4 at a 1% final concentration. For the preparation of the second gel, we employed two polymer stock solutions (1% AC and 36% PlubisSH) that were mixed at a 1:1 ratio, in order to obtain the final concentration of the composite mixture. Both solutions were freshly prepared before experimentations and were stored at 4°C before use.

To form the gels, AC 1% solution and AC 0.5% / PlubisSH 18% solution have to be mixed with the oxidation solution at a 4:1 ratio. This oxidation solution was freshly prepared by combining sodium periodate (NaIO_4_) at 1 mg.mL^-1^ in PBS with a solution of NaOH (0.4 M in PBS) at a 4:1 ratio. The final mixture with AC/PlubisSH solutions and oxidation solution turned light brown and gelled in 2 minutes at 37°C. The 4:1 ratio was selected because it corresponds to a NaIO_4_ concentration known to be non-toxic [29].

### Peptides-hydrogels preparation

Hydrogels containing a final concentration of 200 μM of CTL peptides were prepared. 20 μL of peptides stock solution (500 μM) in PBS were added to 80 μL of AC solution or to 80 μL of the AC/PlubisSH mixture solution described above (40 μL of AC and 40 μL of PlubisSH). Then, 28 μL of oxidation solution were introduced and homogenized to initiate the gelation reaction. The resulting hydrogels were incubated at 37°C for 17h in a humidified incubator. For the control gel (gel without CTL), PBS was used to replace peptides.

#### Sol-gel transition phase diagram

The sol-gel phase transition diagrams for AC/PlubisSH, (with and without oxidation solution) were determined using a vial- tilting method. For this, each solution of hydrogel mixture was prepared in a 2 mL test tube and the temperature was gradually increased with an increment of 1°C. The phase transition temperature was determined once no fluidity was observed after 5 minutes. At each temperature point, samples were equilibrated at least 5 minutes. Mixtures without NaIO_4_ /NaOH were used as a control.

#### Monitoring in vitro hydrogels mass erosion and swelling

*In vitro* hydrogel stability was evaluated by monitoring the mass erosion rate under physiological conditions. 1 mL of each hydrogel was prepared in test tubes and incubated at 37°C overnight for thermal stabilization. Then, 1 mL of PBS (pH 7.4) was added to the hydrogel and the tube was incubated at 37°C. After defined time intervals, the supernatant was removed and the weights of the remaining solid hydrogels were measured. All samples were triplicated.

### Rheological evaluation

Rheological properties of hydrogels were monitored using a rotating rheometer (Thermo Haake Rheowin Rheometer RS-100, Fisher Scientific, France) equipped with a temperature controller. The solid-like behavior (elastic modulus, G’) and liquid-like behavior (viscous modulus, G”) were recorded using two 30 mm parallel plates. 5 mL of hydrogel were applied on one plate to form a thickness of 5 mm.

We used a frequency 1 Hz to capture the gelation time for 15 min of experiments. The crossover point of G’ and G” is then usually considered to be the gelation time [28, 30]. All measurements were replicated 3 times. We also determined the rheological properties of the gels as a function of frequency in the range between 1 Hz and 10 Hz for 1-day-old gels.

### Peptides compound release

Peptide release from gels was estimated using rhodamine labeled CTL (Proteogenix, France). The purity of CTL-rhodamine was evaluated using an automatic Edman sequencing apparatus (Applied Biosystems, France). The hydrogels containing a final concentration of 200 μM CTL-Rhodamine were preparated from 500 μM CTL-Rhodamine stock solution in water. The protocol was described before in the “Peptides-hydrogels preparation” section. The resulting hydrogels were incubated at 37°C for 17h in a humidified incubator. For the control gels (gels without CTL-Rhodamine) water were used to replace peptides. After incubation, 150 μL of MH medium were added on the gels. At a given time, 50 μL of supernatant were transferred on 96-well plates for 570 nm fluorescence (spectrofluorimeter Xenius XC, SAFAS, Monaco). Immediately, another 50 μL of MH medium were added to the gels to maintain a constant volume of supernatant. This step was repeated for each delay decided for an analysis. The fluorescent peptides released from the gels were quantified for 2 days (37°C, PBS).

### Tissue adhesion studies

Tissue adhesion was measured using a force sensor device equipped with a hydrogel holder (MTS Systems, dynamometre, Lhomargy, DY34, France, using the Testworks software). A titanium disc was attached to a cylindrical probe (10 mm diameter) with commercial cyanoacrylate glue. The hydrogel (100 μL) was placed on this disc. After 5 min, the gel was pressed either by another titanium disc (10 mm diameter) alone, or with a piece of attached gingiva of pig (1 cm^2^) glued on a support, for 30 min at 37°C with 0.1 mN of normal force. We collected adhesion data by pulling the probe with a loading rate of 0.3 mm.min^-1^. All measurements were performed in triplicate.

### Bacterial growth

Prior to each experiment, one bacterial colony of *Porphyromonas gingivalis* (ATCC strain 33277) was inoculated overnight under strict anaerobic conditions at 37°C with 10 mL of Brain Heart Infusion (BHI) medium (Sigma Aldrich), supplemented with hemin (5 μg.mL^-1^) and menadione (1 μg.mL^-1^, Sigma Aldrich). The next day, this culture was used to inoculate a second preculture (10% vol. of first preculture). The final bacterial suspension used in our experiments was a mid-logarithmic phase culture of bacteria with a starting optical density of 0.001 (at 620 nm).

### Microbiological tests

#### Bacterial Viability

The experiment consists in the analysis of the bacterial viability in the supernatant of the gels. The Alamar Blue cell viability reagent was employed as a bacteria health indicator (using the reducing power of living bacteria). When the pathogens are alive with a metabolic activity, they maintain a reducing environment in their cytosol. Resazurin, the active ingredient of Alamar Blue assay, is a nontoxic, bacterial permeable compound that is a blue, weakly fluorescent molecule. Living bacteria are able to reduce this molecule to resorufin, a red color, a highly fluorescent molecule (570 nm). The damaged bacteria are not able to reduce the resazurin. For this test, the gels (with or without CTL) were produced in the 96-well plate as described previously. The amount of gel per well was similar for all conditions (100 μL). Then, 100 μL of bacteria suspension (with a OD_620_ equal to 0.001) was added on each well. After incubation, the supernatants were transferred into a 96-well plate. Then, 10 μL of Alamar Blue reagent was added and incubated 3 hours at 37°C. The fluorescence intensity was then monitored at 570 nm.

#### Colony formation assay

These assays evaluate the capacity of entrapped CTL to inhibit bacterial growth in the surrounding media of gels. Each gel is placed in a 96-well plate. 100 μL of bacterial suspension (prepared as described above) is layered on top of each gel. After 5h and 24h of incubation, the supernatant of each type of hydrogel was transferred onto an agar plate. The number of colonies obtained was counted after 24h. The inhibition of colonies number is represented as a percentage of colonies inhibition: (number of colonies formed from the peptides gels supernatant / number of colonies formed from gels control supernatant) x 100.

### Cytocompatibility tests

#### Cell culture

A human line of gingival fibroblasts HGF-1 (ATCC CRL 2014) was used in this study. The cell line was cultured on T-75 culture flasks using culture media supplemented with 10% (v/v) fetal bovine serum (FBS, Gibco, France) and 1% (v/v) antibiotics containing penicilin-streptomicin (Gibco). The cells were incubated at 37°C, 95% relative humidity, and 5% CO_2_.

#### Leachable Assay

The viability of cells on hydrogels was evaluated according to ISO 10993–5 norm. HGF fibroblasts were seeded into each well of a 96-well plate at a density of 1.10^4^ cells/well. After 24h, the medium was replaced with the gel supernatant (100 μl), and then incubated for 3 days. After the incubation period, the extract was replaced with 100 μl of fresh medium containing 10% of Alamar Blue solution. Living cells reduce Alamar Blue reagent in a red color (570 nm). Cells incubated with pure medium (absence supernatant coming from gels) was used as a positive control (live) and cells exposed to a 70% ethanol solution were used as a negative control (dead).

#### Live/Dead observations

The Live Dead Kit (Life Technology, France) is based on a combination of two molecules: the calcein AM and the ethidium homodimer-1 (EthD-1). The polyanionic dye calcein AM labels living cells with an intense uniform green fluorescence. EthD-1, which enters into cells having damaged membranes, provides a bright red fluorescence. Stained cells were observed under epifluorescence microscope illumination (Nikon Eclipse TE200 with 63x PL APO (1.4 NA) objective equipped with Nikon Digital Camera (DS-Q11MC) and with NIS-Elements software, Nikon, Japan).

## Results

### Hydrogel formation

We first investigated the influence of NaIO_4_ on the gel formation of AC/PlubisSH (S2 Fig). The AC concentration was fixed at 0.5 (w/v%) and the PlubisSH concentration was varied. To form a gel at 37°C, a PlubisSH concentration of at least 16% (w/v) is required both in the presence and absence of NaIO_4_. In the absence of NaIO_4_, no gel is formed below this 16% PlubisSH concentration whatever the temperature, whereas in the presence of NaIO_4_ a gel is observed but only at higher temperatures. This is due to the cross-linking actions of NaIO_4,_ in both the chemical reaction and physical gelling. In the subsequent work, we will always use a PlubisSH concentration of 16% (w/v).

**Fig 1:**
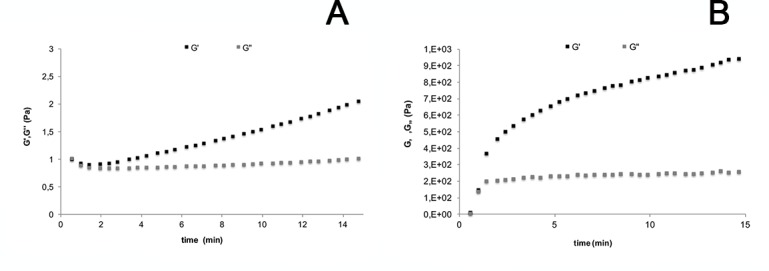
Gelation kinetics of the AC and AC/PlubisSH gels. Rheological properties of AC (A) and AC/PlubisSH (B) hydrogels recorded at a frequency of 1 Hz at a temperature of 37°C (elastic modulus, G’ and viscous modulus, G”).

To determine more precisely the gelation time, we performed dynamic rheological measurements where we determined the storage and loss moduli G' and G" (Fig 1).

Taking the equality of G' and G" as gelation marker we found that AC (1% w/v) and AC/PlubisSH, both supplemented with an oxidation solution, form a gel within the first 2 minutes. As expected, the AC/PlubisSH appeared much more rigid than the AC gel (1000 Pa for AC/PlubisSH compared to 2 Pa for AC for G' at 1 Hz).

### Hydrogel swelling

S3 Fig shows swelling and mass erosion behaviors of the hydrogels in PBS solution, at 37°C and at pH 7.4. AC and AC/PlubisSH hydrogels crosslinked with the NaIO_4_/NaOH are non-degradable for at least 28 days. A weak mass increase was observed for these two gels over 15 days which can be attributed to swelling. In contrast, in the absence of NaIO_4_, the AC/PlubisSH gels started to erode after 12 days. This result shows that the presence of an oxidizing solution is needed to stabilize the gels over a long period of time, as required for clinical applications.

### Adhesion properties

**Fig 2:**
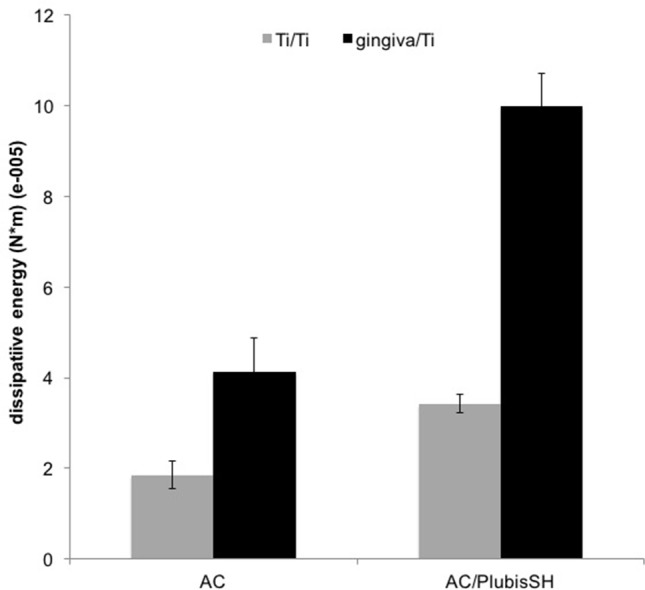
*In vitro* quantitative adhesion of hydrogels on titanium (Ti) and gingiva. Dissipative energy during detachment of AC and AC/PlubisSH hydrogels from gingiva is measured after 30 min of contact with the substrates (Ti/Ti or Ti/gingiva) at 37°C.

We assessed two interfaces in contact with each side of the gel: titanium/titanium (that represents implant-abutment connection), and titanium/connective tissue (that represents abutment or implant junction to gingival soft tissues). Ti_6_Al_4_V, commonly used for the fabrication of dental implants, was used in this study. Pig’s attached gingiva was used to evaluate gel adhesion. As shown in Fig 2, the dissipative energy produced for AC/PlubisSH substrate during detachment is significantly higher than for AC gel, especially for Ti/gingiva interfaces (4 J.m^-2^ compared to 10 J.m^-2^).

### Peptides released from the gels

**Fig 3:**
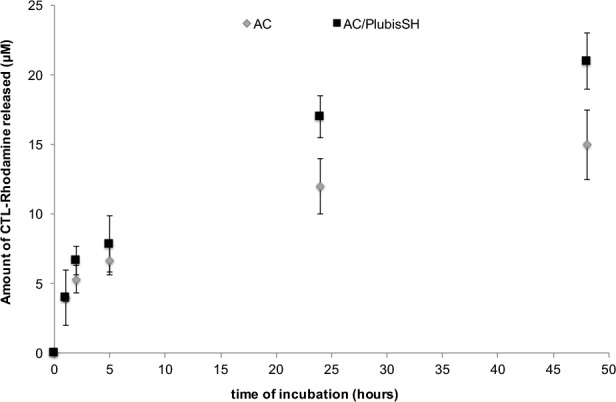
Monitoring of CTL released from the gels. Fluorescence spectrophotometry was used to monitor CTL-Rhodamine released out of AC and AC/PlusbisSH gels over time (MH medium, 37°C).

To follow the release of the CTL, the gels were charged with 200 μM fluorescent CTL-Rhodamine. The delivery of this molecule was monitored for 48h (Fig 3).

The results show that the release takes place at least over 48 hours and that it is greater with the AC/plubisSH gel compared to the AC one. The quantity released after 48 hours for the AC or AC/plubisSH gel is about 15 μM and 20 μM respectively. Thus, about 1/10 of peptides initially loaded into the gels were released after 48 hours. Moreover, we demonstrated in S3 Fig that these two gels are stable over at least 28 days which indicates that some CTL can be probably released during several weeks.

### Antibacterial activity

#### Bacteria metabolic activity

**Fig 4:**
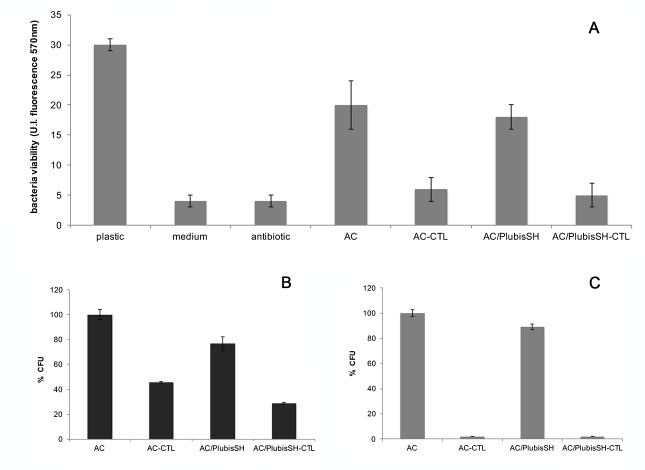
Antibacterial activity of gels. Metabolic activity of bacterial supernatant after 24h (A). « Plastic » corresponds to a negative control, *i*.*e*. bacteria in medium on the 96-well plate, without any gel. « Medium » corresponds to culture media in the 96-well plate without any bacteria or gel. « Antibiotic » corresponds to the positive control with bacteria in medium with two standard antibiotics (tetracyclin and cefotoxim) as supplements. The asterisk (*) denotes a statistical difference between the metabolic activity of *P*. *gingivalis* found in the supernatants of AC and AC-CTL gels, (#) indicates a statistical difference between the metabolic activity of *P*. *gingivalis* found in the supernatant of AC/PlubisSH and AC/PlubisSH-CTL (p < 0.05). Inhibition of colonies forming units (CFU) of the supernatants after 24h of seeding (B and C). These supernatants were previously removed from the gels respectively after 5h (B) and 24h (C) of seeding. The control used in these figures (corresponding to 100% CFU) corresponds to colonies on agar plates obtained from supernatant of AC gel without CTL (Tissue Culture Polystyrene was not used as control because it leads to an homogenous growth of bacteria without colonies). Error bars represent means ± SD.

The metabolic activity of *P*. *gingivalis* was determined after 24 h of incubation by using the Alamar Blue assay. Fig 4A shows that the reducing power of *P*. *gingivalis* cytosol appears to be affected by the peptides released in the supernatant. The bacteria are unable to transform the resazurin in resorufin, indicating that they lost most of their mitochondrial activity.

#### Colonies forming

We assessed the capacity of formation of bacterial colonies by displaying the 5h and 24h gel supernatants on the appropriate agar plates. After 24 h of incubation, the number of colonies was counted. 55% inhibition of colonies formation with AC-CTL and 60% inhibition with AC/PlubisSH-CTL for supernatants incubated for 5 hours are observed in comparison with controls corresponding to similar gels without CTL. For a contact time of 24h, there is a 100% inhibition of colonies formation with AC or AC/PlubisSH gels containing CTL. In contrast, *P*. *gingivalis* present in the supernatant of control gels without CTL are still able to form colonies (Fig 4B and 4C).

### Cellular biocompatibility

The 5h and 24h supernatant solutions (with or without CTL) were kept in contact with the human gingival fibroblasts (HGF) during 3 days. Cell viability was first quantified using the Alamar Blue assay. The results show that the supernatants of the two types of gels (with or without antimicrobial peptides) are not toxic for the human gingival fibroblasts. Their viability (100%) is similar to positive control (cells in the presence of culture media) (S4 Fig).

**Fig 5:**
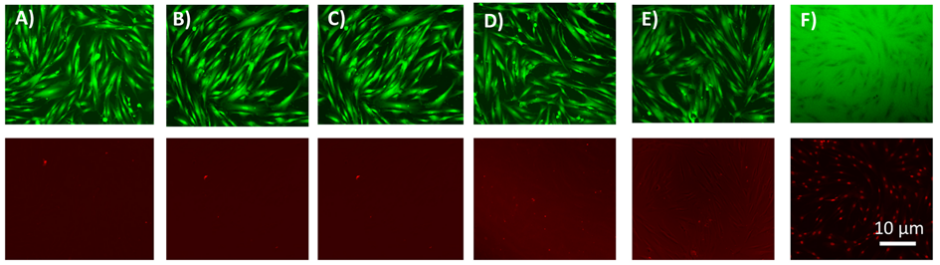
Evaluation of cellular biocompatibility of the gels. (A-F) The LIVE-DEAD observations of cells after 3 days of contact with the 24h gel extract solutions of gels, respectively with AC, AC-CTL, AC/PlubisSH-CTL, ACPlubisSH compared with positive and negative controls. Top pictures in green represent living cells. Images in the bottom row in red represent dead cells.

Then, in a second series of experiments to perform a Live Dead assay, the 24h supernatant solutions of the gels (with or without CTL) were introduced in 96-well plates containing HGF. After 3 days of contact, the cells were stained with Live Dead kit and observed under a fluorescence microscope (Fig 5).

The non-fluorescent calcein is enzymatically transformed in an intensely green fluorescent calcein for the cells that were in contact with the gels extracts, and also for the cells in the positive control experiment, where cells were only in contact with culture medium. As negative controls we brought cells in contact with an ethanol solution. In this case all cells label red. This indicates that the viability of the fibroblasts is not affected by the presence of gel extracts.

## Discussion

The adhesion of mussels in water uses proteins rich in DOPA (3,4-dihydroxy-L-phenylalanine), an amino acid bearing a catechol group. In this project, we used a natural polysaccharide, sodium alginate, modified by catechol groups along the polymer chains. All the gel-forming components are liquid and can easily be injected with a mixing-syringe on the required sites. After mixing, gelation takes place rapidly (within two minutes) in the presence of oxidative reagent (NaIO_4_). Subsequently, the gels can easily be delivered in a preventive way during abutment and implant connection, or later on surfaces of implant and tissues contaminated with periimplantitis bacteria. This catechol-modified alginate (AC) has already been proposed for use in medical applications like treatment of atherosclerotic plaques and has been demonstrated for its efficiency and absence of pro-inflammatory response [29]. In order to improve the mechanical robustness of the gel, we also incorporated pluronic F127 functionalized at both ends with thiol groups (S1 Fig). Pluronic is a commercially available triblock copolymers often used for medical applications related to physical hydrogel formation at 37°C and at physiological pH [31, 32]. After addition of NaIO_4_ the catechol groups strongly interact with the thiol groups to form covalent bonds leading to a covalent network between alginate and pluronics. This is confirmed by demonstrating stability of the gels over more than 21 days at 37°C and increased mechanical storage modulus G'. Moreover, it appears that the addition of pluronics increases the dissipative energy of detachment of the gel from titanium alloys and even more from gingiva as it is required for dental applications. This effect originates most probably from formation of covalent bonds between catechol moieties from gel with amines, thiols, imidazoles residues found in the extracellular matrix proteins and carbohydrates of gingiva.

For the first time, we introduced the antimicrobial peptide CTL into the gels (AC and AC/PlubisSH) with the aim to inhibit *P*. *gingivalis*, the most virulent pathogen of periimplantitis diseases. The CTL antimicrobial peptide was chosen because it is known to be active against a large variety of pathogens, in particular *S*. *aureus* and because it has non-hemolytic properties [12]. In this study, we showed that it also has activity against *P*. *gingivalis* when released from the gels. More precisely, the antibacterial activity of CTL-based hydrogels was evaluated after incubation with *P*. *gingivalis*. The supernatants of the gels with and without CTL contained a similar number of bacteria after 24h (S5 Fig). However, the metabolic activity of the pathogens in contact with the supernatants of the CTL containing gels was strongly reduced (Fig 4A). This result is in accordance with the incapacity of bacteria found in the CTL-gels supernatant to form colonies on agar plates. During these experiments, AC-CTL and AC/PlubisSH-CTL gels exhibited the same antibacterial properties against *P*. *gingivalis*.

Our antibacterial tests clearly demonstrate that CTL has a bacteriostatic effect on *P*. *gingivalis*. We hypothesize that the positively charged CTL peptides released from the gels adsorb at the surface of bacteria and block the negatively charged *P*. *gingivalis* membrane adhesins. It is known also that the adhesins help bacteria in obtaining essential nutriments for growth and survival [33]. In a previous paper by using Surface Plasmon Resonance Spectroscopy it was demonstrated that human beta-defensin 3 binds to hemagglutinin B, a non fimbrial adhesin from *Porphyromonas gingivalis* (K_a_ = 1.80x10^4^) and attenuates a pro-inflammatory cytokine response [34]. The authors report that the binding of human beta-defensin 3 to hemagglutinin B may be related to its high cationic charge. In this context, we suppose that cateslytin with its net positive charge of +5 might also interact with hemagglutinin B.

In our case, blocking the *P*. *gingivalis* adhesins (fimbrili or pilli) shows indirectly an inhibition of mitochondrial enzymatic activity that will block bacteria division [34]. Thus, we suggest that the mechanism used by the CTL to inhibit the metabolism of *P*. *gingivalis* is based on the « charged lipid clustering model », one of the AMP’s mechanism proposed by Nguyen et al. [35]. In this model, AMP interacts with the lipid membrane to achieve an antimicrobial activity but without disruption of the membrane.

The biocompatibility of the gels was estimated with human gingival fibroblasts in the presence of gels supernatant. All gels demonstrate excellent fibroblast proliferation after 3 days of incubation, indicating no obvious signs of toxicity.

## Conclusion

Injection of the AC or AC/PlubisSH gels between implant and abutment or other medical device would act as a physical preventive barrier, limiting the development of bacteria. Moreover, introduction of CTL in these gels inhibits *P*. *gingivalis* development in the surrounding living environment, controlling virulence factor seeding. The presence of PlubisSH in the gel enhances mechanical and adhesive properties, making these gels an ideal candidate to manage peri-implant sites in a preventive way. Further investigations will explore other applications of these gels and their utility in patient treatments.

## Supporting Information

S1 Fig Chemical structure of the catechol-modified Alginate (A) and the thiolated Pluronic F-127 (B). (TIF)

S2 Fig Sol-gel transition curve of the gels. Curve without gelling solution (A); and in presence of oxidation solution (B).
(TIF)

S3 Fig Mass erosion of hydrogels. Assay conduced in PBS, at 37°C, monitored for 1 month.
(TIF)

S4 Fig Viability of the human gingival fibroblasts in presence of the 5h and 24h supernatant gels solutions. (A) Percentage of cells viability for AC and AC-CTL gels; (B) Percentage of cells viability for AC/PlubisSH and AC/PlubisSH-CTL gels.
(TIF)

S5 Fig Evaluation of total number of bacteria found in the supernatant (OD 620 nm). Bacterial supernatant is in contact with gel for 24h.
(TIF)
