# Supplementary figures and images for: Antibacterial Peptide-Based Gel for Prevention of Medical Implanted-Device Infection
Source: PLoS One. 2015 Dec 14;10(12):e0145143. doi: 10.1371/journal.pone.0145143 (PMC4682826; doi:10.1371/journal.pone.0145143)

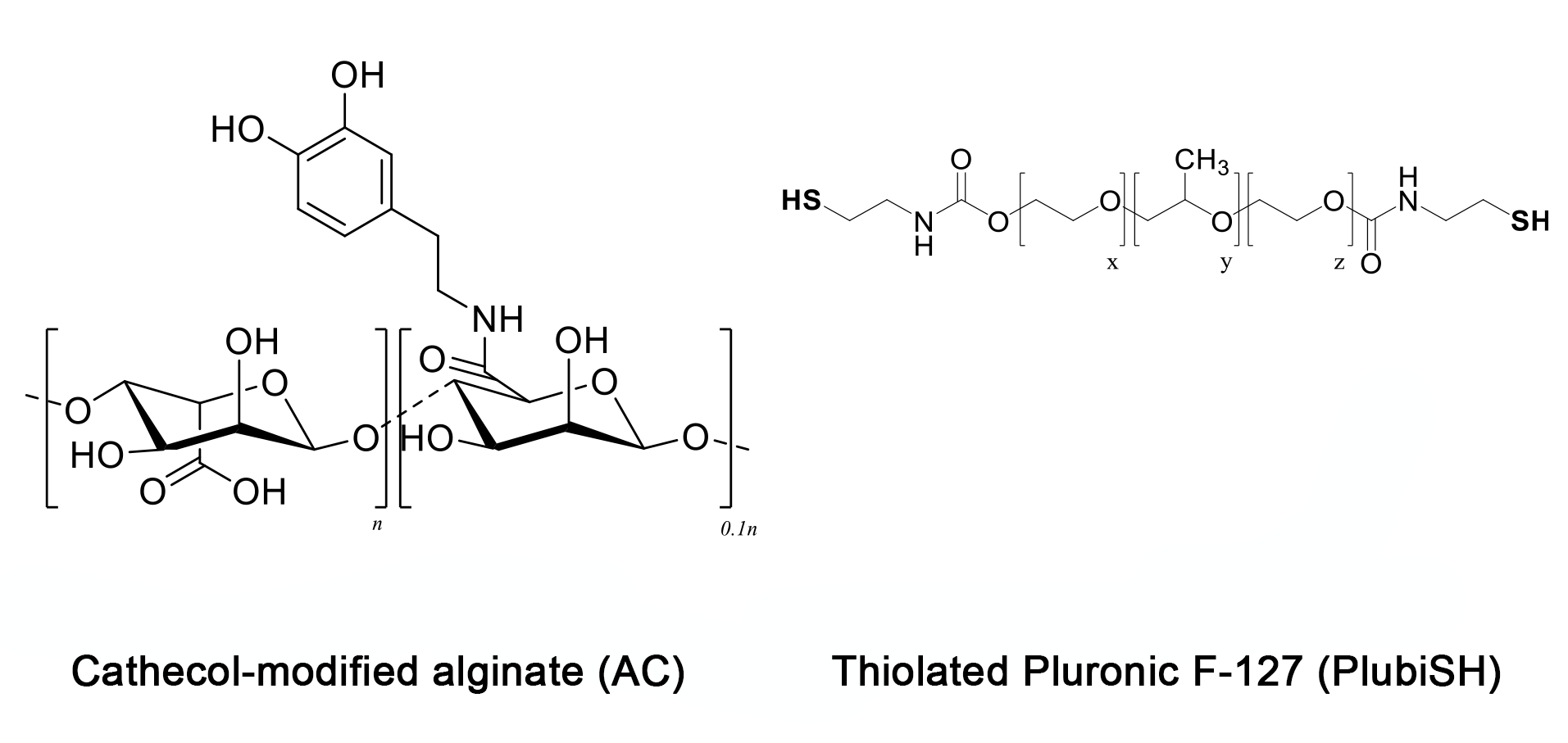

Supplement: S1 Fig — (TIF) [file pone.0145143.s001.tif]

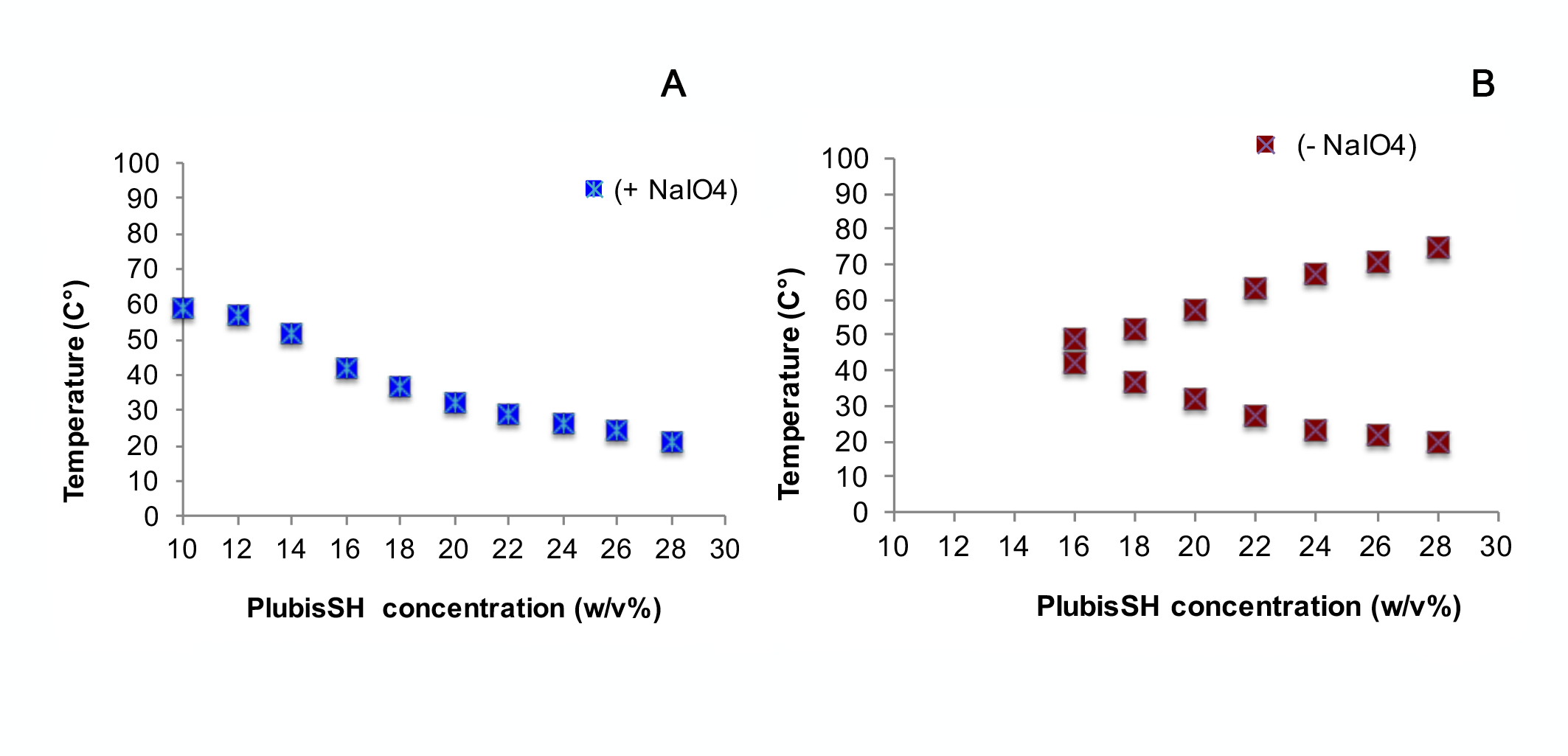

Supplement: S2 Fig — Curve without gelling solution (A); and in presence of oxidation solution (B). (TIF) [file pone.0145143.s002.tif]

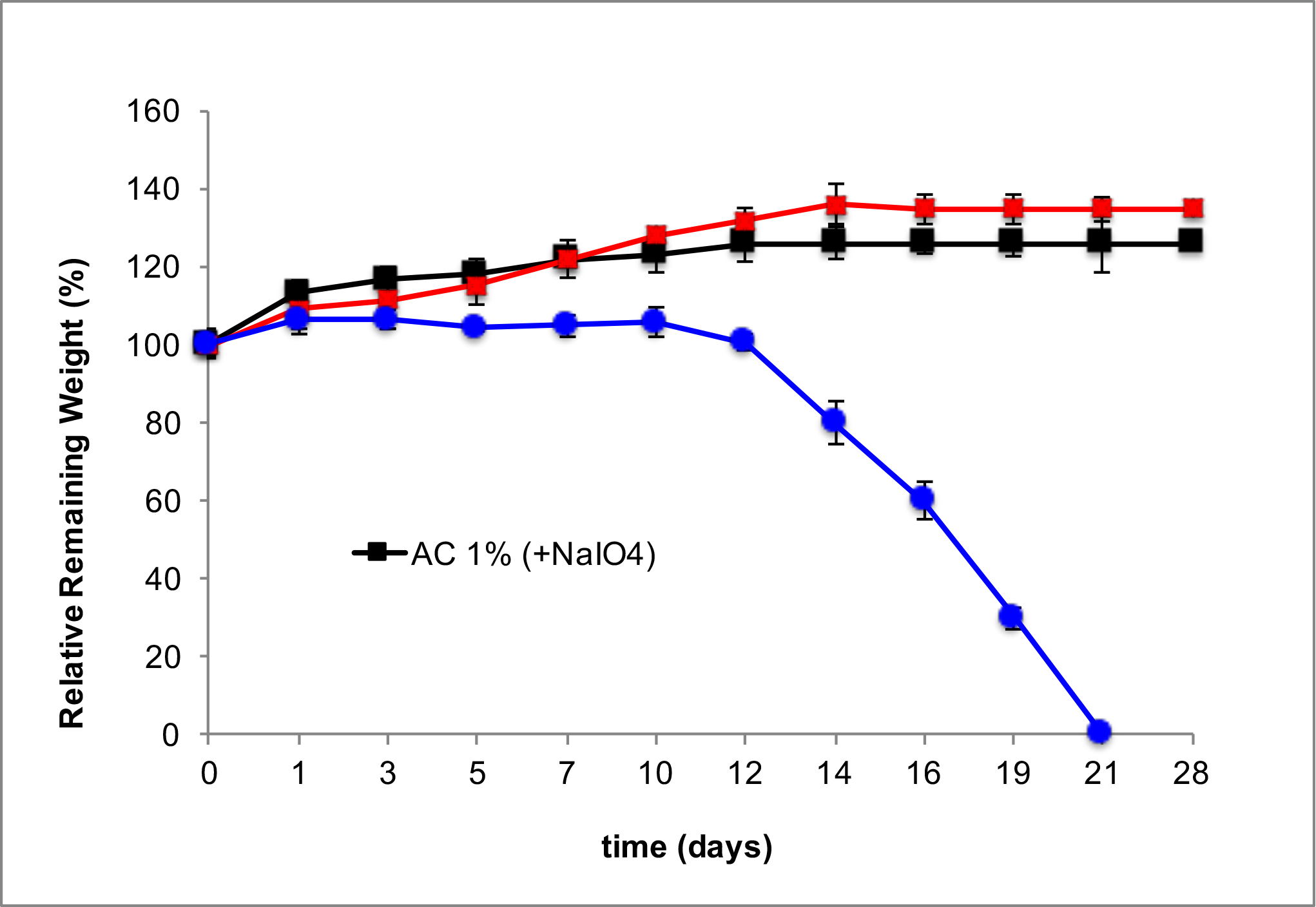

Supplement: S3 Fig — Assay conduced in PBS, at 37°C, monitored for 1 month. (TIF) [file pone.0145143.s003.tif]

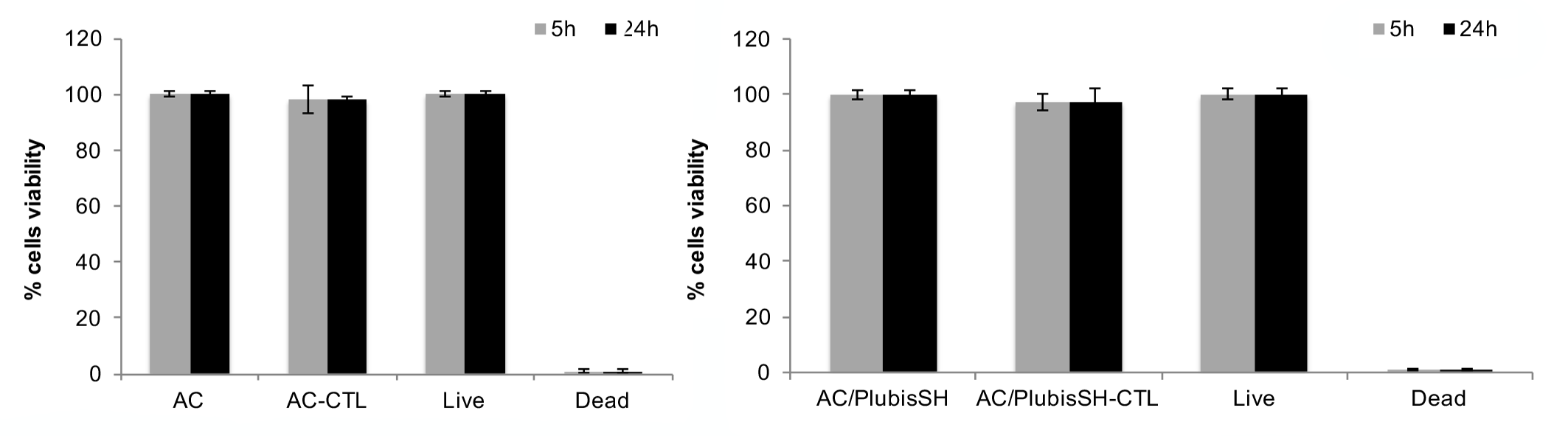

Supplement: S4 Fig — (A) Percentage of cells viability for AC and AC-CTL gels; (B) Percentage of cells viability for AC/PlubisSH and AC/PlubisSH-CTL gels. (TIF) [file pone.0145143.s004.tif]

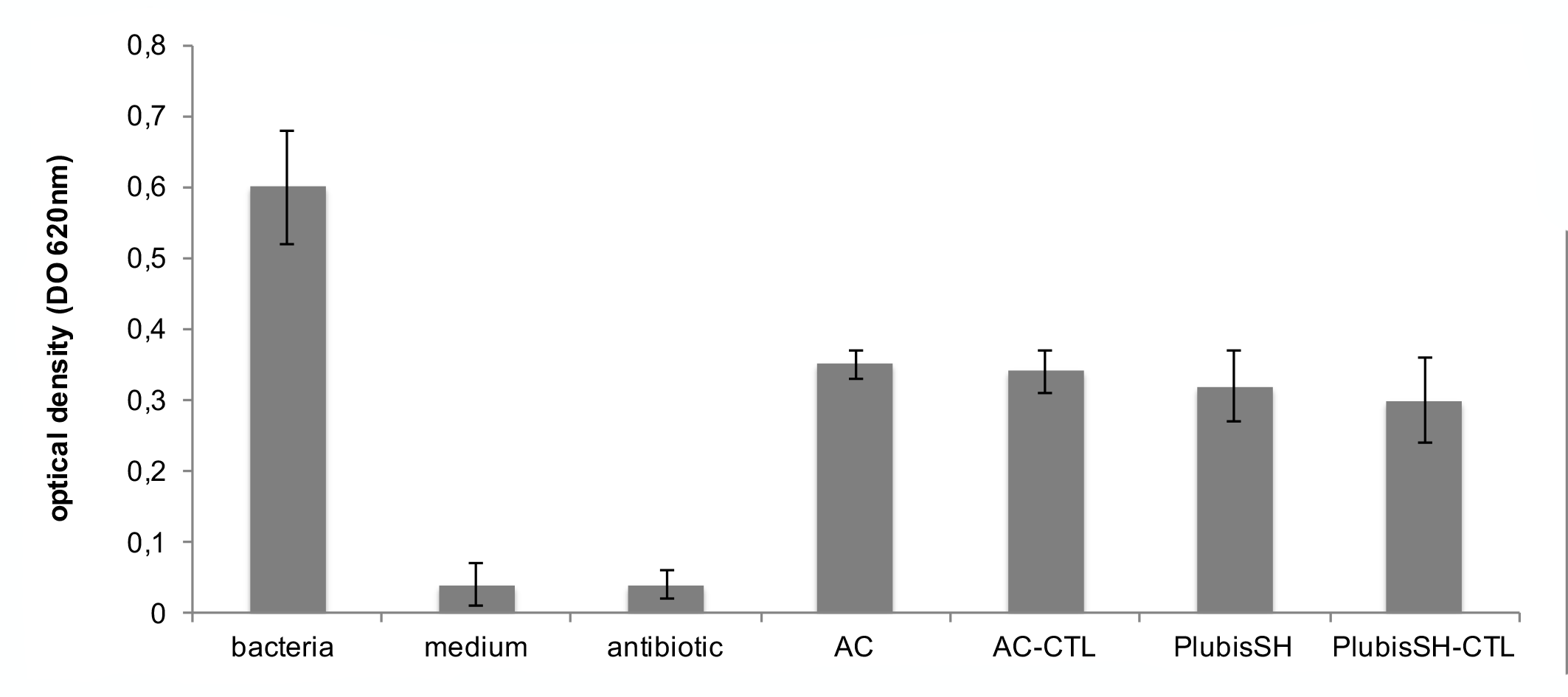

Supplement: S5 Fig — Bacterial supernatant is in contact with gel for 24h. (TIF) [file pone.0145143.s005.tif]
